# Digital Social Norm Enforcement: Online Firestorms in Social Media

**DOI:** 10.1371/journal.pone.0155923

**Published:** 2016-06-17

**Authors:** Katja Rost, Lea Stahel, Bruno S. Frey

**Affiliations:** 1 Institute of Sociology, University of Zurich, Zurich, Switzerland; 2 Center for Research in Economics, Management and the Arts, Zurich, Switzerland; University of South Australia, AUSTRALIA

## Abstract

Actors of public interest today have to fear the adverse impact that stems from social media platforms. Any controversial behavior may promptly trigger temporal, but potentially devastating storms of emotional and aggressive outrage, so called online firestorms. Popular targets of online firestorms are companies, politicians, celebrities, media, academics and many more. This article introduces social norm theory to understand online aggression in a social-political online setting, challenging the popular assumption that online anonymity is one of the principle factors that promotes aggression. We underpin this social norm view by analyzing a major social media platform concerned with public affairs over a period of three years entailing 532,197 comments on 1,612 online petitions. Results show that in the context of online firestorms, non-anonymous individuals are more aggressive compared to anonymous individuals. This effect is reinforced if selective incentives are present and if aggressors are intrinsically motivated.

## Introduction

Collective online aggression directed towards actors of public interest is an increasing phenomenon. While various types of social media have been involved in such online firestorms (e.g. content communities such as YouTube), blogs and social networking sites such as Facebook are outstanding triggers [1]. In 2011, Christian Wulff, the former federal president of Germany, was accused of corruption–claims that afterwards were rejected as unfounded although they promptly led to his resignation. The Wulff-affair was massively amplified by the negative word-of-mouth dynamics in social media. In 2013, the company Amazon was accused of the ill treatment of temporary workers. The Amazon-affair led to floods of negative comments on Amazon’s Facebook profile. Firestorms also shake academia: In 2011, the former minister of defense of Germany, Karl-Theodor zu Guttenberg, was accused of plagiarism. These accusations triggered widespread online debates and ultimately led to the denial of his PhD and to his resignation.

The examples illustrate how online aggression has emerged from the private niche of limited email bullying to a publicly visible and relevant phenomenon. Dependent on the focus of the underlying research, the phenomenon of aggressive, offensive and emotional commenting in social media has been labeled flaming, cyberbullying, online harassment, cyber aggression, electronic aggression, toxic online disinhibition, trolling or, if the aggression resembles crowd-based outrage, online firestorms [1–5]. In online firestorms, large amounts of critique, insulting comments, and swearwords against a person, organization, or group may be formed by, and propagated via, thousands or millions of people within hours [1]. Social media enable these unleashed phenomena [2, 3, 6]. They allow attacking everywhere at anytime with the potential for an unlimited audience. They raise the likelihood for hostile misinterpretations due to limited discursive action and social media’s absence of nonverbal cues. They reduce the risk for feedback reactions because users can “sneak off” after the aggressive act.

The phenomenon of online aggression is not well understood despite the great deal of attention on hostile behavior in social media in both the mainstream media and the empirical literature [2, 7–16]. Most contributions are descriptive and are conducted largely in the absence of theories [2, 15]. If contributions refer to theories they are mainly guided by traditional bullying research theory, more precisely by the massive amout of existing research concerned with cyberbullying among adolescents. Within this view, online aggression is understood as an irrational and illegitimate behavior that is caused by underlying personality characteristics, such as a lack of empathy and social skills, narcissism, impulsivity, sensation seeking, emotional regulation problems or psychological symptoms such as loneliness, depression, and anxiety [15, 17]. Traditional bullying research theory, however, misses the point that in online firestorms, aggression happens in public, and not in private, social networks.

It therefore seems questionable whether bullying research theory is transferable to online firestorms. For example, a strong and commonly shared assumption within bullying research theory is that anonymity, understood as the degree to which a communicator perceives the message source as unknown and unspecified, promotes aggression through decreased inhibitions [3, 18–21]. For online firestorms it suggests that negative, and particularly aggressive, word-of-mouth propagation in social media will weaken if real-name policies are introduced. In this article we show that this assumption is not necessarily true because the reverse effect can be obtained: Individuals have a strong motivation for being non-anonymous when being aggressive in social media. We explain this behavior pattern by social norm theory. Social norm theory may be a more appropriate theory to understand communication behavior in social media and to draw conclusions, for example, that real-name policies will not weaken online firestorms.

The remainder of this paper is structured as follows: the next section introduces social norm theory to understand aggressive behavior in a social-political online setting, and develops hypotheses. The subsequent sections explain the dataset, the measurements and the method, and present the empirical findings. We conclude with a discussion of the findings, research limitations and suggestions for further research.

### A social norm theory on online firestorms

Social norms are fundamental to human behavior [22, 23]. Former literature defines norms as statements “that something ought or ought not to be the case” ([24] page 132), as institutionalized role expectations [25], or as becoming apparent if behavior attracts punishments [26]. In general, norms are mental representations of appropriate behavior in society and smaller groups and, consequently, guide the behavior of individuals. Norms that are characterized as social “must be shared by other people and partly sustained by their approval and disapproval” ([23] page 99). Social norms are created intentionally because they promote the provision of a public good that benefits a collective, for example less pollution in a neighborhood due to less burning of leaves [27], less harm to health through cessation of smoking [28], or more fairness through income differentials [29, 30]. The public good view does not automatically imply that social norms are always beneficial for all persons concerned. In fact, many social norms exclude certain groups from public goods because they promote the interest of one subgroup, i.e., they serve “functions of inclusion and exclusion” ([23] page 108). For example, peer-group norms aim to strengthen cohesion within the group by offering group privileges [23, 31].

To be sustainable, social norms need to be enforced, otherwise Olson’s [32] zero contribution holds: “if all rational and self-interested individuals in a large group would gain as a group if they acted to achieve their common interest or objective, they will still not voluntarily act to achieve that common or group interest” ([32] page 2). Social norms are enforced by simple sanctions which trigger feelings of guilt and shame in the case of internalized social norms. Consequently, the mere expectation of sanctions, in turn, supports the enforcement [23]. Enforcement also happens through actual bilateral and multilateral costly sanctions where those who cause negative externalities are confronted with punishments and normative demands [28, 33]. Linked to Olson’s [32] zero contribution, norm enforcement itself is a second-order public good: self-interested and utility-maximizing individuals do not naturally contribute to norm enforcement and may prefer free riding [28, 33]. Ostrom [34] however stresses how, in practice, contextual variables and the engagement of certain types of individuals determine whether collective action and cooperation is enhanced or discouraged. Similarly, Ellickson [35] emphasizes how norms may emerge or shift dependent on cost-benefit conditions or group composition. Also the presence, salience, or strength of social ties can explain individual variation in social-political engagement [36, 37]. For example, diffuse networks of weak bridging ties encourage mobilization through communicative advantage [38]. Specifically, research shows that Olson’s [32] second-order public good dilemma can be overcome if (1) norm enforcement is cheap, i.e., it occurs in low cost situations [27, 39], (2) additional benefits are provided to the norm enforcers that disproportionately motivate them compared to non-enforcers, i.e., selective incentives are present [28, 32] and/or (3) if some individuals are present that are intrinsically motivated to enforce norms, i.e., some amount of altruistic punishment occurs [40–42]. In the following we elaborate these three conditions for social media to explain the phenomenon of online firestorms.

### Online firestorms within a social norm theory

Aggressive word-of-mouth propagation in social media is the response to (perceived) violating behaviors of public actors. Public actors include, for example, politicians who disregard political correctness norms, corporations that violate human rights, or academics who violate scientific norms by engaging in plagiarism. In this view, online firestorms enforce social norms by expressing public disapproval with the aim of securing public goods, for example, honesty of politicians, companies or academics. The stunning waves of aggression typical for online firestorms can be explained by the characteristic features of social media that ideally contribute to the solution of the second-order public good dilemma of norm enforcement. Digital norm enforcement in social media is cheap, and selective incentives and intrinsically motivated individuals are present.

In social media, sanctioning norm violations occurs in low-cost situations. The basic idea of the low-cost hypothesis is that attitudes or preferences are more likely to guide individual behaviors when norm enforcement behavior is relatively cheap [27, 39, 43]. Evidence in various research fields supports this basic tenet (for an overview see [43]). For example, the voting paradox [32], i.e., the fact that citizens participate in elections even though they are aware of the marginal influence of their vote, is often explained by referring to the low-cost hypothesis [44]. In social media, a number of factors contribute to such low-cost situations. First, social media mobilize former free riders because online criticism is monetarily inexpensive, hardly time-consuming and can be performed anywhere and anytime, compared, for example, to elaborate street protests [1, 2]. One example is the limited message length in the social media platform Twitter, which obliges communication to be short and quick. It is less astonishing that Twitter has been involved in most of the recent cases of online firestorms [1]. Second, in social media, people who are geographically completely removed from each other can assault each other verbally without fear of bodily harm. Nonverbal cues such as facial expression and physical size are lacking, thus reducing the empathy of the aggressor and the impact of authority of the victims typically expressed by dress, body langugage, and social setting [2, 3, 45]. Third, social media give ordinary people the power to communicate (perceived) norm violations to a very large audience [46, 47]. The internet re-creates village-like interconnectedness within a global, pluralistic society by crossing local, or even national, boundaries due to unrestrained information flow [48]. To compare, while aggressive norm enforcement is a rare behavior in the non-digital context (Brauer and Chekroun [49] found that max. 4% of bystanders aggressively sanction daily deviant behavior by insulting or aggressive shouting), we should observe it more frequently in the digital social media context for the reasons given above.

#### Hypothesis 1

Provided that a social-political issue finds its way into social media platforms, online aggression takes place more frequently than in the non-digital context because sanctioning of (perceived) norm violations occurs in low-cost situations.

In social media, selective incentives that benefit a latent group of norm enforcers are disproportionally present [28, 32]. Individuals only bear the costs of norm enforcement if the potential benefits of their actions exceed the costs [50]. Selective incentives translate resentment for norm breaching into action in situations where it is unclear whether a necessary critical mass of other norm enforcers will join the action. In such situations, cost sharing cannot be expected, nor can clear benefits from norm enforcement, such as an actual behavioral change by the accused person or organization, be predicted. In the case of selective incentives, individuals participate in collective action in response to salient private benefits [51]. Whether individuals are able to reap selective incentives is dependent on the issue at stake and on certain individual or group characteristics. Social media contribute to the presence of selective incentives by enhancing the salience of private benefits. In social media, for example, highly controversial topics are debated. Social media are, in addition, highly influenced by the multiplication of cross-media dynamics, for example by public scandals taken up or created by news media leading to comments in social media. Broad public discussions and connections to public scandals give credible signals that a norm infringement at the expense of a latent interest group–be it the group an individual belongs to or identifies with–has occurred [52].

#### Hypothesis 2

Online aggression in social media is encouraged by salient selective incentives, for example, in highly controversial topics or in topics connected with public scandals.

Social media ensure that a high amount of intrinsically motivated actors are present. Individuals engage in costly norm enforcement if they have an intrinsic desire to “make the world a better place” [53–55]. This type of norm enforcement has been intensively discussed as “altruistic punishment”, i.e., individuals punish, although the punishment is costly for them and yields no material gain [42]. Altruistic punishment is driven by strong negative emotions towards the norm defector [40, 41, 56] and by people’s perception of a state of affairs as illegitimate [57–61]. Strong intrinsic motivation, however, is only likely to encourage participation if it is reinforced by organizational or individual ties [37]. This requirement is given in the infrastructural setting surrounding online firestorms. The technical mechanisms of social media such as newsletters, newsgroups, followers, or social media sharing ensure that intrinsically motivated individuals are optimally informed about cases that, in their view, represent offenses against existing social norms. Beyond this, they provide opportunities to tackle these norm violations by commenting on them.

#### Hypothesis 3

Intrinsically motivated actors encourage online aggression in social media.

### The non-anonymity of negative word-of-mouth dynamics in social media

In social media, people can hide or alter their identity. They may either comment by providing no name or at least not their real name, i.e., a (random or stable) pseudonym. Existing literature on online behavior hypothesizes that such online anonymity is one of the principle factors that decreases inhibitions, increases self-disclosures and therefore promotes online aggression [3, 18–21]. This causal mechanism is also assumed by social media consultants who attempt to explain online firestorms [62].

In general, anonymity produces the “stranger on a train” phenomenon, wherein people share intimate self-disclosures with strangers as they do not expect a reunion and hence do not fear any risks and constraints [63]. To that effect, “when people have the opportunity to separate their actions online from their in-person lifestyle and identity, they feel less vulnerable about self-disclosing and acting out” ([3] page 322). With regard to heightened aggression and inappropriate behavior, psychosocial motives exist for being anonymous [19]. Anonymity first detaches from normative and social behavioral constraints [64]. Second, it allows to bypass moral responsibility for deviant actions [3]. Third, it reduces the probability of social punishments through law and other authorities [20]. Fourth, it triggers an imbalance of power which limits the ability of the victim to apply ordinary techniques for punishing aggressive behavior [65]. Fifth, it gives people the courage to ignore social desirability issues [3] and finally, it encourages the presentation of minority viewpoints or viewpoints subjectively perceived as such [66–70].

Former research has concluded that the possibility for anonymity in the internet fosters aggressive comments. It is assumed that online aggression is driven by lower-order moral ideals and principles and, consequently, people feel ashamed to aggress under their real names. However, the empirical evidence for such a link is scarce and no definitive cause-effect relationship has evolved. Studies suggest that anonymity only increases online aggression in competitive situations [71], that anonymity does not increase online aggression but does increase critical comments [72], or that the effect of forced non-anonymity on the amount of online aggression is a function of certain characteristics of user groups, e.g. their general frequency of commenting behavior [73].

The former conceptualization of online aggression is rather narrow, in particular for aggression in social media. According to social norm theory, in social media, individuals mostly use aggressive word-of-mouth propagation to criticize the behavior of public actors. As people enforce social norms and promote public goods, it is most likely that they perceive the behavior of the accused public actors as driven by lower-order moral ideals and principles while that they perceive their own behavior as driven by higher-order moral ideals and principles. From this point of view there is no need to hide their identity.

Furthermore, aggressive word-of-mouth propagation in a social-political online setting is much more effective if criticism is brought forward non-anonymously. This is due to the fact that non-anonymity inceases the trustworthiness of the masses of weak social ties to which we are linked, but not necessarily familiar with, in our digital social networks. Trustworthiness of former firestorm commenters encourage us to contribute ourselves. First, non-anonymity is more effective as the credibility of sanctions increases if individuals use their real name [70, 74]. Anonymity makes “information more suspect because it [is] difficult to verify the source’s credibility” ([70] page 450). This removes accountability cues and lets one assume that individuals present socially undesirable arguments [74, 75]. Second, the views of non-anonymous individuals are given more weight: “Just as people are unattached to their own statements when they communicate anonymously, they are analogously unaffected by the anonymous statements of others” ([69] page 197). Anonymous comments have less impact on the formation of personal opinions [69, 76], on the formation of group opinions [74], and on final decision making [77]. Third, anonymity lowers the identification with, support of, and recognition by, kindred spirit [78]. In anonymous settings, individuals cannot determine who made a particular argument, how many different people expressed similar arguments, whether a series of arguments are all coming from the same person, or the degree to which other commenting individuals are similar to oneself [74, 79–81]. Anonymity filters out cues that communicate social identity, cues that are necessary to characterize comments by others [74, 82], to identify with individuals in social comparison processes [74] and to coordinate group interactions [80]. Finally, anonymity reduces the benefit to be positively evaluated by others [83, 84]. Studies show that exclusively anonymous conditions induce little mobilization because anonymity excludes the benefit of recognition by others [85].

From a social norm point of view, the arguments suggest that aggressive word-of-mouth propagation in a social-political online setting takes place non-anonymously. People have a strong feeling to stand up for higher-order moral ideals and principles. Commenting anonymously is a costly, wasteful behavior, as sanctions are less credible, create less awareness, less support and offer few benefits. These considerations make particular sense in the usual setting of firestorms, namely social media where usually, weak social ties are clustered around ideologically like-minded networks. Such networks likely support non-anonymous aggressive sanctions that confirm their worldview.

#### Hypothesis 4

In a social-political online setting, non-anonymous individuals, compared to anonymous individuals, show more online aggression.

As stated earlier, norm enforcement is fostered if selective incentives and intrinsically motivated actors are present. Consequently if social norm theory is an appropriate theory for online aggression in a social-political online setting, these groups in particular should give more weight to the benefits of non-anonymous aggressive word-of-mouth propagation. Simultaneously, they give less weight to potential risky consequences such as being subject to deletion, banned from websites, formally convicted by the accused actor for defamation of character and/or damage to reputation, or informally sanctioned by social disapproval from online or offline individuals [86].

#### Hypothesis 5

In a social-political online setting, in situations that offer selective incentives, compared to situations without selective incentives, more online aggression by non-anonymous individuals is observed.

#### Hypothesis 6

In a social-political online setting, intrinsically motivated aggressors (i.e. aggressive commenters), compared to aggressors without intrinsic motivation, show more online non-anonymous aggression.

## Materials and Methods

### Sample

We test the hypotheses with a census of a major social media platform concerned with public affairs. We analyze all comments on online petitions published at the German social media platform www.openpetition.de between May 2010, the launching of the online portal, and July 2013. Online petitions exemplarily include protests against pay-scale reform of the German society for musical performing and mechanical reproduction rights called GEMA (305,118 signers), against the enforcement to finance public service media (136,010 signers), against the closing of the medical faculty at the University Halle (58,577), or for the resignation of an Austrian politician (9,196 signers) or the Bavarian minister of justice (6,810 signers). Online petition platforms seem very suitable to investigate the phenomenon of negative word-of-mouth in a social-political online media setting. First, online petitions are concerned with public actors and public affairs, for example, internet security, misbehavior of firms, politicians, or academics, public spending, tax issues, animal protection, etc., and thus provide a central location where public norms are negotiated. Second, online petition platforms are prototypical social media platforms: everybody is allowed to participate and create content for any kind of topic, and the debates and comments are publicly visible. Third, qualitative evidence suggests that many popular firestorms have been triggered or have been surrounded by online petition platforms, for example the Deutsche Telekom firestorm in 2013, or the firestorm leading to the displacement of the German Federal President Christian Wulff in 2011. Fourth, online petition platforms are concerned with real-life cases. Many former studies are based on artificial laboratory experiments to study negative word-of-mouth behavior on the internet. Finally, online petition platforms cover a wide range of public issues and affairs, implying lower selection biases as compared to case studies about online firestorms (such as in [1]).

The final dataset includes 532,197 comments on 1,612 online petitions. There were a total of 3,858,131 signatures over the 1,612 petitions between 2010 and 2013, with detailed information about the wording of the comment, the commenters, the signers and the petition. The dataset was provided to the authors in an anonymous form by the platform owner. For each signer and commenter, however, the dataset indicated whether he/she had originally contributed anonymously (= 1) or non-anonymously (= 0). For this study, no approval of any ethics committee was sought because all data are publicly accessible on www.openpetition.de and no names of signers or commenters can be tracked and identified in the dataset. In order to prepare the dataset in accordance with our theory, we rely on a mixed-method big-data approach. For many variables we use a qualitative approach to arrive at meaningful quantitative measurements.

The present dataset allows us to exclude two biases which, in other studies, frequently affect findings on relations between anonymity and aggression. First, there was no active intervention in the ratio of anonymous and non-anonymous aggressive comments in the dataset. In the period of data collection, the platform owner did not moderate the comments on his own initiative. However, he reacted by deleting selected inappropriate comments when the user community reported them. According to the platform owner, a deletion was independent of whether the inappropriate comment was provided anonymously or not, as he explicitly considered this difference as irrelevant to liability issues. Second, we may also exclude any bias stemming from differing legal jurisdictions: Potential legal implications for identified aggressors are the same across the entire study. In Germany, the jurisdiction on defamation and insult is part of the federal law [87], i.e., as the entire study pertains to the same legal jurisdiction, all defamatory or aggressive commenters across all German states face the same potential costs for their actions.

### Measurement of Variables

We measure online aggression in the following manner. In general, inconsistency in the operationalization of online aggression dominates research [88]. Operationalization includes impolite statements, swearing, flirting, exclamations, expressions of personal feelings, use of superlatives [89] to profanity, typographic energy (e.g. exclamation marks), name calling, swearing, and general negative effect [72, 88]. We rely on the definition of online aggression in firestorms, i.e., large amounts of critique, insulting comments, and swearwords against a person, organization, or group formed by, and propagated via, social media platforms [1]. Accordingly, we measure online aggression by direct offenses within the comments on online petitions (e.g. “I hate GEMA, complete morons and exploiters”, ID469090), swearwords (e.g. “Fuck that Shit!”, ID477368), and expressions of disgust or contempt (e.g. “The deportation policies of German authorities is commonly a disgusting, repulsive and inhuman mess!”, ID418089). Expressions of disgust and contempt are typical responses to morally offensive behavior [90]. Importantly, even from the outside perspective, we confidently evaluate these expressions to be intended as aggression. This is because we do not expect close relationships or shared, subcultural interactional norms between the commenter and the targeted actor in petitions, in contrast to profane language between friends representing covert closeness and not aggression [91].

To systematically collect online aggression, we compile a list of frequently used swearwords from synonym reference books and online databases of swearword collections (e.g. http://www.schimpfwoerter.de/). This approach corresponds to previous studies that count aggressive postings by using a pre-defined set of aggressive words (such as in [73]). Then, we disaggregate the 532,197 comments into single words and count them. Frequently occurring words are manually checked and classified as online aggression if applicable. Subsequently, we exclude all words that can be used for different meanings, for example, as swearwords or as terms for animals. These steps led to a final list of 1,481 words that express offenses, swearwords, and disgust. Using this final list of aggressive expressions, we count the amount of online aggression in each comment. Subsequently we qualitatively check the appropriateness of our approach by comparing subsamples of comments with our quantitative measurement. We take the logarithm added by 1 to create an approximate normal distribution of the variable.

#### Independent variables

Anonymity is measured in the following way: Before online users sign a petition and subsequently formulate a voluntary comment, they are requested to provide their real names and addresses. In regard to public visibility, they are given the choice to allow their real name to be published or to remain anonymous, i.e., only the postal code is visible to other users (0 = non-anonymous, 1 = anonymous). Although the theoretical possibility of using pseudonyms does exist, we expect that commenters’ incentive for pseudonyms is low. This is because anonymity complies with the hidden name option and petition organizers may classify the signature of pseudonyms as invalid.

Controversy that accompanies a petition is measured by the level of debate. Each petition provides the opportunity to start a debate on the petition homepage, a tool used in most petitions by supporters and opponents. A debate is structured by denoted pro- and contra-arguments, i.e., by arguments that underpin or oppose the petition’s concerns. Only arguments that differ in their content from formerly mentioned arguments are additionally incorporated. Within the pro- and contra-sections, commenters are allowed to oppose arguments by adding sub-replies (pro-reply-/contra-reply-arguments). More controversial topics lead to a higher diversity of pro-, contra-, pro-reply- and contra-reply-arguments. Thus, to measure controversy, we construct a Herfindahl index by taking the percentage of arguments within each category, i.e., pro-/contra-/pro-reply-/contra-reply-arguments, squaring it, adding them together and subtracting the final result from 1. The index measures the controversy that surrounds the topics of petitions from no controversy (= 0) to a maximum of controversy (= 1).

To identify scandals, we measure whether the accusation against an actor forwarded by a petition, for example corruption of a politician, is covered and framed as scandal by traditional news media (1 = yes / 0 = no). We define keywords that describe the content and concerns of the petition. In the database LexisNexis we search for whether these keywords are associated with the term “scandal” in the German-speaking media within a time period of one year before the starting date of each petition.

To measure actors’ intrinsic motivation, we operationalize fairness perceptions of commenters. We compile a list of 579 expressions frequently used in ideological discourses that indicate fairness issues, for example, expressions such as “injustice” or “unfair”. In addition, we use synonym reference books and databases, manually check frequently occurring words within comments and exclude ambiguous words. For each commenter we count the amount of intrinsic motivation by taking the sum of fairness words in the comment. We take the logarithm, added by 1, to create an approximate normal distribution of the variable.

#### Control variables

We control for factors that influence the amount of online aggression.

The length of comment is measured by the total number of words in a comment. Longer comments are more likely to entail more aggression.

The time period between opening a petition and submitting a comment is included because the time point of comment submission may influence commenters’ level of aggression. Aggression may either take place in the very beginning, because most signatures and comment activity in petitions are submitted within the first days [92], or alternatively, in advanced stages, in the case where a petition experiences a boost due to revived public debate. We measure how many minutes after petition opens that a comment has been submitted.

The number of protesters having signed is included because larger protests are likely to attract more online aggression. We measure how many individuals sign a particular petition and consequently match this data with the comments on a certain day. The median of protesters amounts to 76 signers per day with a maximum of 2,926 signers per day. We take the logarithm of the number of protesters to create an approximate normal distribution of the variable.

The status of the accused may also influence online aggression. Theoretically, public actors with a high social status may be either protected from sanctions as they have more resources to reply to punishments by even more painful punishments, or, to the contrary, they can attract sanctions because they are also more vulnerable than lower status actors [93]. In practice, high status celebrities or politicians may also refrain from suing laypersons as it is counterproductive to their reputation. To take these complex influences into account, we control for the status of the accused. As a proxy for social status of the accused public actors, we collect the number of Google hits for the accused’s name (1 = <1000; 2 = <10,000; 3 = <100,000; 4 = <500,000; 5 = <1,000,000; 6 = >1,000,000). Google hits tend to reflect social status. To decrease measurement errors, for example due to actors sharing the same name, we additionally check whether the accused is listed in the German online encyclopedia Wikipedia (0 = no entry, 1 = entry in article’s subtitle, 2 = entry as main article). Wikipedia exclusively lists actors with a minimum public status. We add both variables and take the logarithm of the mean value.

We measure also whether the accused is a natural person or a legal entity. Legal entities professionally monitor the internet for defamation and gather more resources to fight accusations than do natural persons. To avoid that commenters anticipate differing costs for their aggressive behavior dependent on whom the accused actor is, we control for this factor. Two independent coders manually check whether the target is a natural person such as a scientist or politician (= 1) or a legal entity such as a government or an organization (= 0). In 4% of the petitions, the target is a natural person and not a legal entity.

The anonymity of the social environment of commenters measures the anonymity of the environment in which commenters live. This may influence how much aggression is expressed [94]. Less anonymous villages with tight social control likely increase sanctioning costs. As a proxy for the anonymity of commenters’ social environment, we measure the size, i.e., the number of inhabitants, of the city or village in which commenters live. The postal codes of each signer are aggregated such that individuals living in the same city or village are merged. The dataset includes 23,977 cities and villages. We count the number of signers for each city or village, and by random checking, we find that the correlation of the number of signers within a postcode region, and the de facto size of this region, is 0.92, validating our proxy. We allocate the size of residence variable to all signers and commenters. Bigger values indicate that commenters originate from more anonymous environments.

The regional scope of a protest is measured because issues of broad public relevance may attract more aggression. We measure the regional diversity of a petition by constructing a Herfindahl index ranging from no regional diversity (= 0) to a maximum of regional diversity (= 1). Signers are assigned to different German federal states on the basis of residential postal codes. We take the percentage of signers within each federal state, square it, add them together, and subtract the final result from 1.

The success of a petition is measured because successful petitions potentially deal with more relevant topics, which may indirectly influence the amount of online aggression. A petition is considered successful if the petition initiator defines the petition goals to be achieved in full or at least in part (1 = yes; 0 = no).

The petition motive may influence the amount of online aggression. Using a petition’s title and leading text, two independent coders classify the petitions with regard to their underlying motives by using the classification by Reiss [95]. Five major concerns are identified, namely idealism/fairness (42%), income/costs (19%), security/social order (13%), autonomy/self-determination (14%), and quality of life/competences (52%). Multiple assignments of petitions are possible. Idealism/ fairness serves as the reference group in the regression models.

Similarly, the petition topic may influence anonymity considerations and the amount of aggression. Depending on the societal area, be it the economy, politics, or culture, accused actors may differ in their thresholds of wanting to sue aggressive online commenters. Commenters may anticipate these thresholds and the related differing costs of being aggressive. This in turn affects commenters’ actual behavior. Using a petition’s title and leading text, two independent coders classify the petitions with regard to their underlying topics using the functional systems of a society [96]. Six major topics are identified, namely society (41%), arts (20%), economics (13%), politics (8%), media (8%), and environment and animal protection (8%). Multiple assignments of petitions are avoided. Society, including topics such as sport or solidarity, is the most general category and serves as reference group in the regression models.

For the summary of the descriptive statistics and bivariate correlations of the former variables, see S1 Table.

### Methods

We apply random-effects and fixed-effects models to predict online aggression in petitions (for access to data, syntax, and Permission for using data of openpetition.de, see the Data availability statement). In both models the comments are grouped on the petition level. The random-effects model keeps within- and between-petition variation in the analysis. We assume that petitions vary not only within, but also between, each other, for example because some petitions have many supporters while other petitions have only a few supporters, or because of differences in the underlying goals and motives. We analyze whether online aggression within and between petitions changes when other variables within and between the petitions change. The fixed-effects model keeps only within-petition variation in the analysis. We also analyze whether the aggression within petitions changes when other variables change, for example the anonymity of commenters, the amount of intrinsic motivation or the amount of selective incentives within the petitions. Many variables of our dataset are time-invariant, i.e., constant petition features that do not vary on the petition level. In the fixed-effects model these variables are omitted. Both models have advantages as well as disadvantages. The fixed-effects model excludes all random noise between the petitions and is therefore often preferred as the golden standard. However, differences between the petitions, for example the number of supporters, may also be important in explaining negative word-of-mouth behavior within petitions. This speaks in favor of the random-effects model. We therefore apply both models and compare the results. We additionally run alternative conceivable models for the data structure, for example, logistic regression, Poisson regression, or negative binomial regression for panel data, as our dependent variable is (if not transformed) a count variable, or can be transformed into a binary variable that indicates whether a person is an aggressor or not. The results are similar with the results that follow and will therefore not be presented here.

## Results

**Fig 1:**
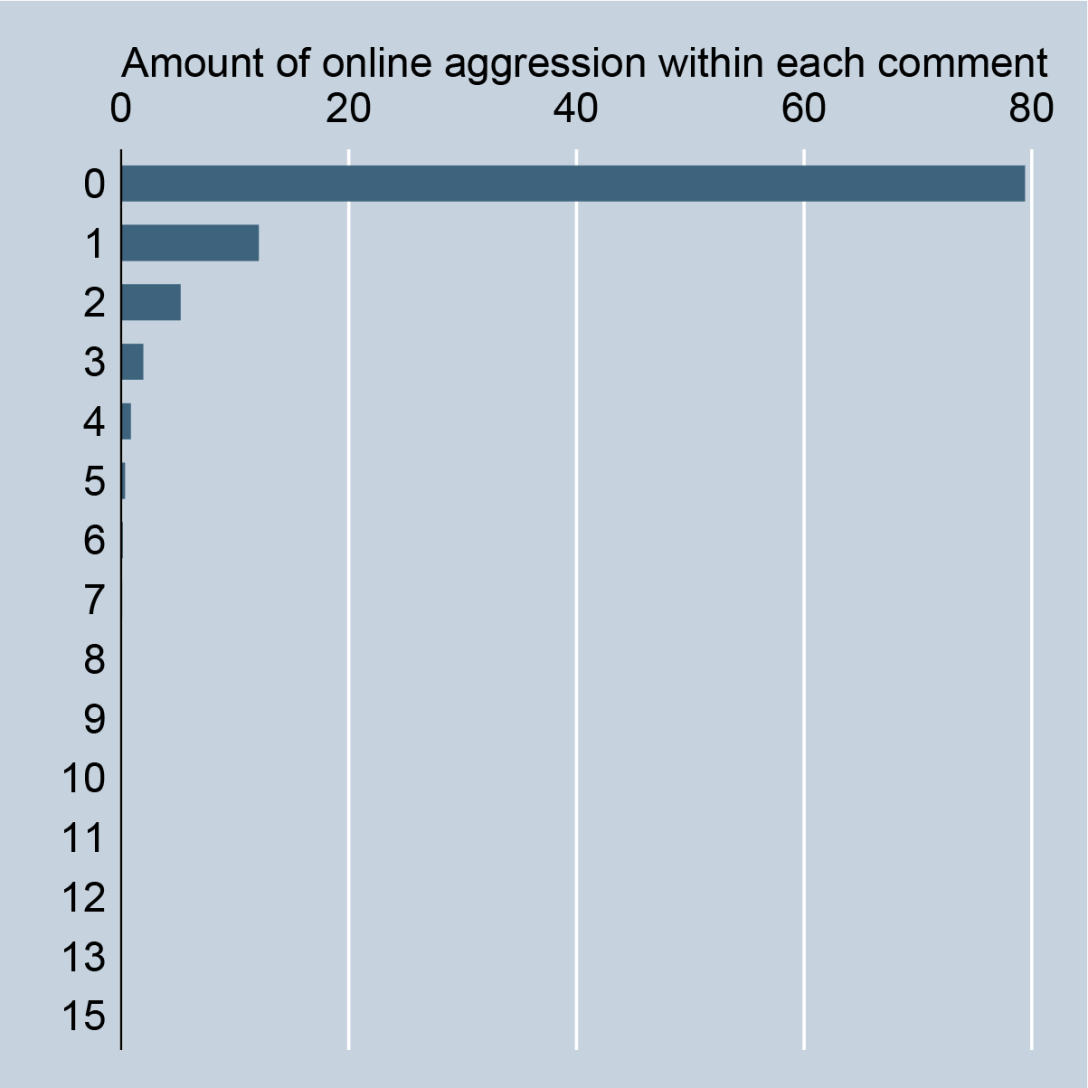
Observed amount of online aggression per comment.

In accordance with Hypothesis 1, the data substantiate that online aggression in social media is a more frequent phenomenon than in the non-digital context. In the analyzed online petition platform we find 197,410 aggressions according to our definition. 20.62% of all comments entail a minimum of one aggressive expression (Fig 1). In 9% of all comments we find two, up to fifteen, aggressive expressions. On the petition level, only 11% of all petitions include no aggressions. 34% include a negligible amount of aggressions from 1, up to 10. 37% include 11 up to 100 aggressions. 16% include 101 up to 1,000 aggressions. 2% include 1,001, up to 25,360, aggressions. Even if the prevailing majority of commenters make no use of aggressive language in social media, the numbers demonstrate that online aggression occurs not only in a vanishing minority of comments or petitions (compared to the observed vanishing minority of max 4% of bystanders aggressively sanctioning in the non-digital context [49]). This supports the claim that in social media, aggressive sanctioning behavior is a relatively frequent phenomenon because it takes place in low-cost situations.

We now move to the presence of selective incentives and intrinsically motivated actors in social media. The descriptive findings show that 47% of all petitions are accompanied by a highly controversial debate, 6% of the petitions are associated with a scandal in news media, and 26% of the commenters are motivated by fairness concerns. Social media thus indeed seem to offer an environment in which the second-order public good dilemma of norm enforcement can be overcome. Whether these conditions indeed contribute to norm enforcement is tested in Tables 1 and 2.

**Table 1:** Predicted amount of online aggression dependent on the anonymity of aggressors (random-effects regression).

	Model 1				Model 2			
Y: Amount of online aggression (log)	Coef.	Std.Err.	z	P>|z|	Coef.	Std.Err.	z	P>|z|
Anonymity	-.02	.00	-13.10	***	.00	.00	-.35	
Controversy of accusation	.04	.01	4.45	***	.05	.01	4.86	***
Accusation is connected to a scandal	.02	.01	2.16	*	.03	.01	2.38	*
Intrinsic motivation (log)	.01	.00	12.17	***	.02	.00	12.15	***
Anonymity x Controversy					-.02	.01	-3.01	**
Anonymity x Scandal					-.01	.00	-3.00	**
Anonymity x Intrinsic motivation					-.01	.00	-3.19	**
Length of comment in words	.00	.00	114.09	***	.00	.00	114.13	***
Time of comment after petition opening	.00	.00	-3.31	**	.00	.00	-3.30	**
Number of protest participants (log)	.00	.00	-.35		.00	.00	-.33	
Scope of protest	.03	.01	3.38	**	.03	.01	3.39	***
Success of the petition	.01	.01	.71		.01	.01	.70	
Status of the accused (log)	.00	.01	-.38		.00	.01	-.43	
Accused is a natural person (vs. legal entity)	.05	.01	4.03	***	.05	.01	4.03	***
Anonymity of social environment of aggressors (log)	.00	.00	-5.69	***	.00	.00	-5.68	***
Motives: Income/minimization of costs	-.01	.01	-1.28		-.01	.01	-1.30	
Motive: Security/social order/traditional values	.01	.01	1.29		.01	.01	1.29	
Motive: Independence/self-determination	.00	.01	.05		.00	.01	.05	
Motive: Increasing life quality and competence	-.06	.01	-8.65	***	-.06	.01	-8.69	***
Topic: Art/culture/education	-.01	.01	-1.25		-.01	.01	-1.26	
Topic: Economics	.02	.01	1.97	*	.02	.01	1.98	*
Topic: Politics	.00	.01	.13		.00	.01	.15	
Topic: Media	.05	.01	4.01	***	.05	.01	4.01	***
Topic: Environmental and animal welfare	.05	.01	4.37	***	.05	.01	4.40	***
Constant	.06	.02	3.88	***	.06	.02	3.70	***
Number of observations			532196				532196	
Number of groups			1568				1568	
R-square (between)			12.69%				12.70%	
Wald chi2			15031.07	***			15066.10	***

Legend

†< p .1

*< p .05

**< p .01

***< p .001

**Table 2:** Predicted amount of online aggression dependent on the anonymity of aggressors (fixed-effects regression).

	Model 1				Model 2			
Y: Amount of online aggression (log)	Coef.	Std.Err.	z	P>|z|	Coef.	Std.Err.	z	P>|z|
Anonymity	-.02	.00	-13.14	***	.00	.00	-.29	
Controversy of accusation	(drop.)				(drop.)			
Accusation is connected to a scandal	(drop.)				(drop.)			
Intrinsic motivation (log)	.01	.00	11.79	***	.02	.00	11.82	***
Anonymity x Controversy					-.02	.01	-3.07	**
Anonymity x Scandal					-.01	.00	-3.00	**
Anonymity x Intrinsic motivation					-.01	.00	-3.18	**
Length of comment in words	.00	.00	114.00	***	.00	.00	114.04	***
Time of comment after petition opening	.00	.00	-3.63	***	.00	.00	-3.64	***
Number of protest participants (log)	.00	.00	-.31		.00	.00	-.29	
Status of the accused (log)	(drop.)				(drop.)			
Scope of protest	(drop.)				(drop.)			
Success of the petition	(drop.)				(drop.)			
Accused is a natural person (vs. legal entity)	(drop.)				(drop.)			
Anonymity of social environment of aggressors (log)	.00	.00	-5.79	***	.00	.00	-5.77	***
Motives: Income/minimization of costs	(drop.)				(drop.)			
Motive: Security/social order/traditional values	(drop.)				(drop.)			
Motive: Independence/self-determination	(drop.)				(drop.)			
Motive: Increasing life quality and competence	(drop.)				(drop.)			
Topic: Art/culture/education	(drop.)				(drop.)			
Topic: Economics	(drop.)				(drop.)			
Topic: Politics	(drop.)				(drop.)			
Topic: Media	(drop.)				(drop.)			
Topic: Environmental and animal welfare	(drop.)				(drop.)			
Constant	.11	.00	33.16	***	.11	.00	32.90	***
Number of observations			532196				532196	
Number of groups			1568				1568	
R-square (within)			2.70%				2.70%	
F-value			2449.47	***			1636.62	***

Legend

†< p .1

*< p .05

**< p .01

***< p .001

**Fig 2:**
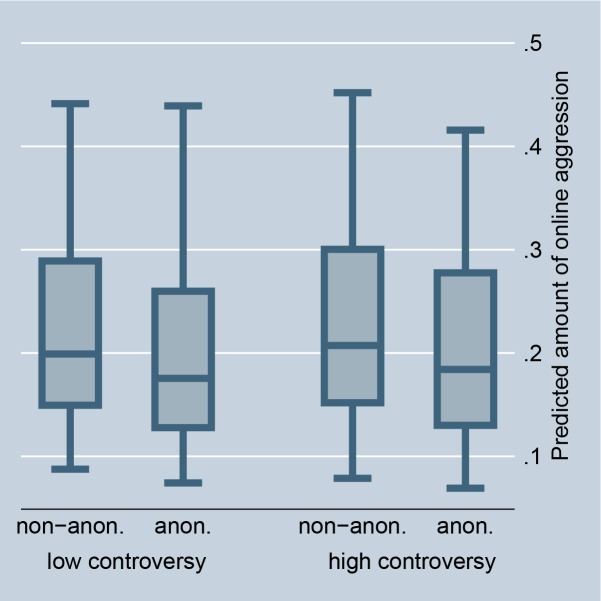
Online aggression dependent on controversy and anonymity (random-effects). Predictions of Table 1, Model 2.

**Fig 3:**
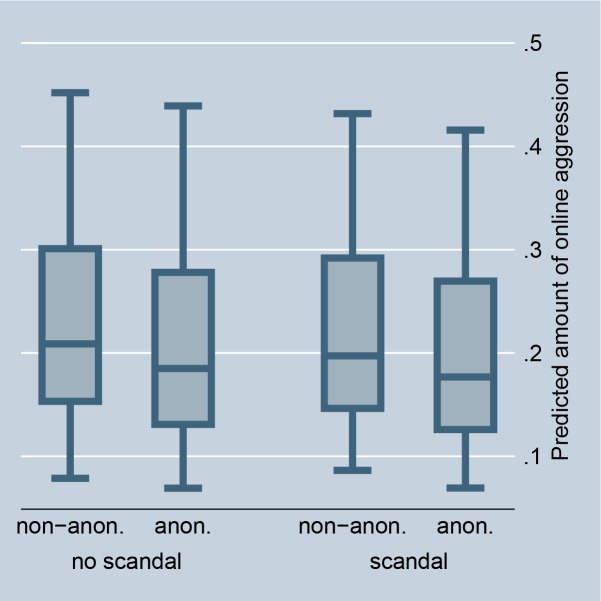
Online aggression dependent on scandal and anonymity (random-effects). Predictions of Table 1, Model 2.

**Fig 4:**
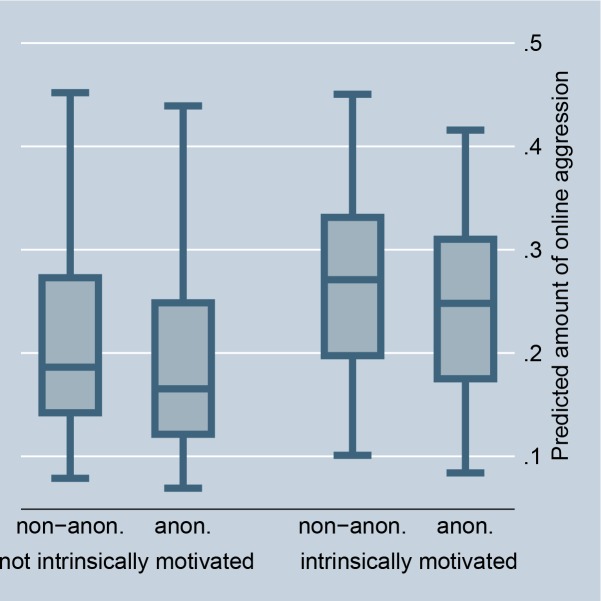
Online aggression dependent on intrinsic motivation and anonymity (random-effects). Predictions of Table 1, Model 2.

**Fig 5:**
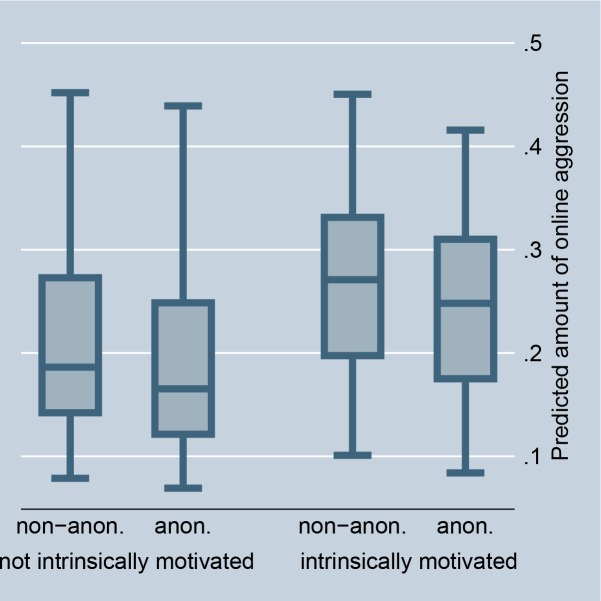
Online aggression dependent on intrinsic motivation and anonymity (fixed-effects). Predictions of Table 1, Model 2.

The random-effects model in Table 1, Model 1, confirms that situations that offer selective incentives, i.e., a petition is accompanied by a highly controversial debate or is connected with a scandal in news media, significantly encourage online aggression in comments. This preliminarily supports Hypothesis 2 (for the size of the effects see Figs 2 and 3). The fixed-effect model in Table 2 entails no results for selective incentives because petition-invariant effects are dropped. Further, the random-effects as well as the fixed-effects models in Tables 1 and 2, Model 1, preliminarily support Hypothesis 3: online aggression is encouraged by intrinsically motivated actors as compared to individuals without fairness concerns (for the size of the effects see Figs 4 and 5).

Building on the view that social media today are a major channel for digital social norm enforcement, which until now is not rejected by the data, Hypothesis 4 assumes that online aggression takes place non-anonmously. Aggressive commentors have nothing to hide: they stand up for higher-order moral ideals and principles. The goal of norm enforcement can be reached most effectively if sanctions are forwarded non-anonymously because they are credible, create awareness, support, and offer benefits. The descriptive statistics show that only 29.2% of all commenters prefer to remain anonymous. Anonymity of commenters is thus a characteristic feature of social media; however, a vast majority still comments under their real names. The results in Tables 1 and 2, Model 1, show the impact of commenters’ anonymity to predict online aggression in comments. In line with Hypothesis 4, both the random-effects and fixed-effects models show that more online aggression is obtained by non-anonymous commenters and not by anonymous commenters.

Exemplarily, we present three of the most aggressive comments by non-anonymous commenters: “Silly, fake, inhuman and degrading, racist, defamatory and ugly theses like those of Sarrazin (author's note: a former German politician) have no place in this world, let alone in the SPD (author's note: Social democratic party). Sarrazin certainly has no business in the Social democratic party and should try his luck with the Nazis” (ID352216); “HC Strache (author's note: Austrian politician) has an evil, inhuman character, he lies and tries to persuade other people of wrong ideas.” (ID284846); “These authorities are mostly no people, but §§§- and regulatory machines! I detest authorities–with my 67 years’ life experience after all!” (ID418089).

**Fig 6:**
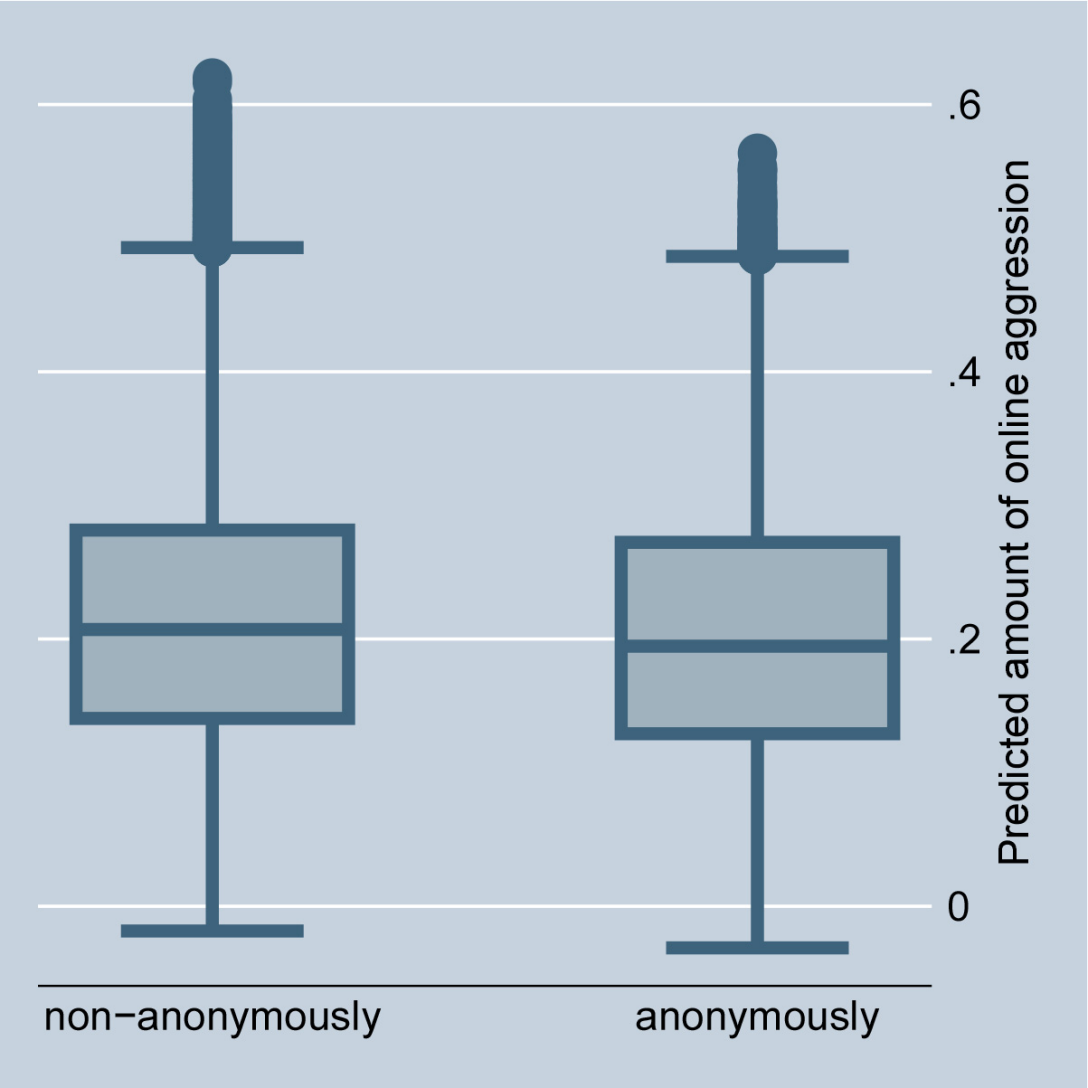
Online aggression dependent on anonymity of commenters (random-effects). Predictions of Table 1, Model 1.

**Fig 7:**
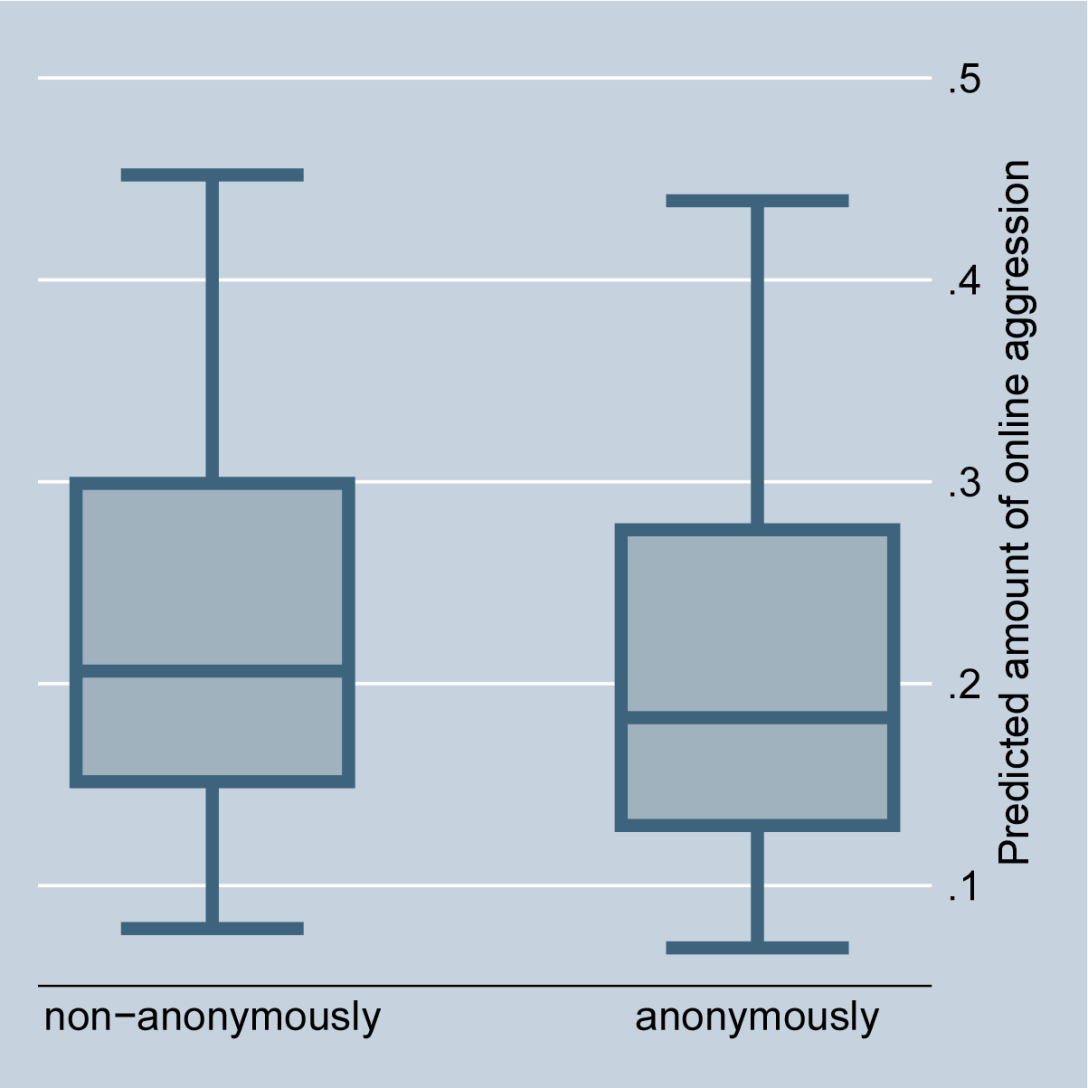
Online aggression dependent on anonymity of commenters (fixed-effects). Predictions of Table 2, Model 1.

Figs 6 and 7 illustrate the size of the effect as predicted in the random- and fixed-effects regressions. The average effect of anonymity on aggression becomes sharper in the fixed-effects model. The random-effects model additionally illustrates that many of the very aggressive commenters appear non-anonymously. Overall, the effect size is small. However, the data clearly show that social norm enforcement, and not as popularly assumed, the risks of detection, seems the major motivation for aggression in social media because persons often aggress under their real names.

If norm enforcement is indeed the major motivation for aggression in social media, the highest amount of non-anonymous negative word-of-mouth should be obtained in situations that offer selective incentives and for intrinsically motivated actors. Model 2, in Tables 1 and 2, tests this assumption by introducing interaction effects between the anonymity of commenters and the presence of selective incentives and their intrinsic motivation. The results give preliminary support for Hypotheses 5 and 6. The highest amount of non-anonymous aggression is observed if a petition is accompanied by a highly controversial debate, is connected with a scandal in news media, and if persons are motivated by fairness concerns. By introducing these interaction effects, the main effect of anonymity on online aggression becomes insignificant, and thus suggests that the underlying reasons for non-anonymous aggression can be indeed explained by social norm theory, namely by selective incentives and intrinsic motivation.

**Fig 8:**
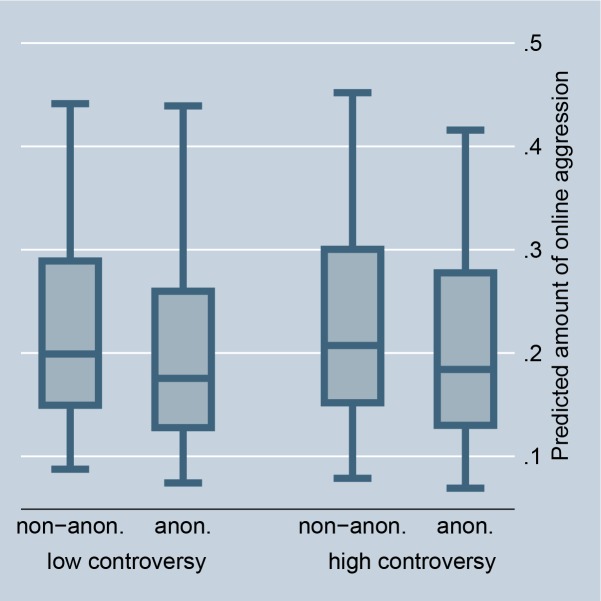
Online aggression dependent on controversy and anonymity (fixed-effects). Predictions of Table 2, Model 2.

**Fig 9:**
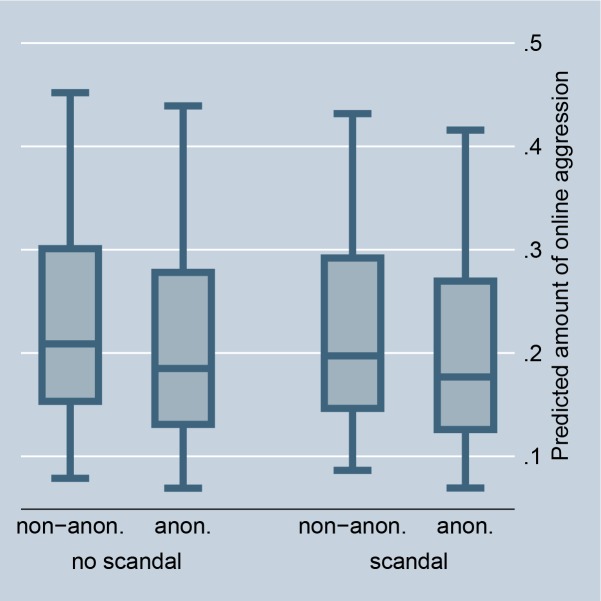
Online aggression dependent on scandal and anonymity (fixed-effects). Predictions of Table 2, Model 2.

Figs 2 and 8 illustrate the effect for the level of controversy within a debate. In the case of highly controversial topics, individuals clearly prefer to aggress non-anonymously, indicating that selective incentives are present (we code debates as highly controversial if the Herfindahl index is higher than 0.3, and as less controversial if the Herfindahl index is 0.3 or smaller). Figs 3 and 9 illustrate the effect for the connection with a scandal in news media. Particularly for scandalized topics, the biggest gap arises between the aggression of non-anonymous and anonymous commenters, with the former showing more aggression. Again it supports that scandals offer selective incentives for norm enforcement. Finally, Figs 4 and 5 illustrate the effect for intrinsically motivated individuals. Intrinsically motivated individuals clearly prefer to aggress non-anonymously.

With respect to the control variables, the results show that longer comments and comments submitted earlier in the process of a petition entail a significantly higher amount of aggression. The daily number of protesters has no effect on the amount of aggression, rejecting the assumption that larger petitions attract more negative word-of-mouth. Online aggression significantly increases for geographically dispersed protests, indicating more general relevance, and for natural persons. Individuals show more online aggression if they live in small villages and cities. We can only speculate about the reasons for this unexpected finding. One explanation is Putnam’s [97] hypothesis that suggests that political participation, and thus also norm enforcement in social media, decrease in large, anonymous regions with a low amount of social capital. Petitions that deal with quality of life entail a significantly lower amount of aggression, whereas petitions that deal with the economy, the media, and environmental or animal welfare entail a significantly higher amount of aggression.

Overall, the random-effects model predicts online aggression by 13%, suggesting that 36% of the variance of aggression can be explained. The fixed-effects model, in which the predictive power is always substantially lower, predicts online aggression by 3%, suggesting that 16% of the variance of aggression can be explained. The predictive power of both models seems rather moderate. One should, however, bear in mind that the predictions are based on objective data, thus implying that common-method biases (and thus systematic-error variance) are absent.

## Discussion

In online firestorms, large amounts of critique, insulting comments, and swearwords against actors of public interest are propagated in social media within hours. This article begins the investigation on this rather new phenomenon by introducing a novel view on online aggression in social media. Relying on social norm theory, we proposed and demonstrated that one major motivation for online aggression in social media is the enforcement of social norms, be it, for example, the struggle for social justice by insulting greedy managers and politicians, or the angst about foreign infiltration by hate speeches against migrants. Norm enforcers punish actors of public interest who cause negative externalities for society or their sub-group by negative word-of-mouth. The technical conditions in social media, such as enhanced visibility and lowered sanctioning costs, have contributed to the expansion of bilateral and multilateral aggressive sanctions which can lead to firestorm-like patterns. Based on this theoretical conceptualization, we also underpinned that online anonymity does not promote online aggression in the context of online firestorms. There are no reasons for anonymity if people want to stand up for higher-order moral principles and if anonymity decreases the effectiveness of sanctions for norm enforcement.

By showing this, we hope to make a number of valuable contributions to the field of online aggression in social media. First, online aggression in a social-political online setting is not primarily an illegitimate and irrational behavior, performed by narcissistic and impulsive actors with a lack of empathy, social skills and emotional regulation problems acting out of personal revenge (such as in [5, 13]). Online aggression in social media resembles a practice of sousveillance [98]: it accomodates a growing digital civil society that actively uses the available masses of weak ties in social media to publicly enforce social-political norms. Social norm theory offers a theoretical foundation for research on online aggression, which up to now has been largely driven by the absence of theory or psychological interpretations of traditional bullying theory (for example [15]). Second, it is one of the first studies that has investigated the role of anonymity for online aggression in a social-political online setting by relying on a large dataset that is representative of the proposed digital civil society, i.e., commenters who actively contribute to a wide range of social-political norm enforcement (see also [73]). Third, we challenged the popular claim that negative word-of-mouth in social media is mainly caused by commenters’ anonymity. In contrast, the results support the idea that non-anonymous aggressive sanctions are more effective. Non-anonymity helps to gain recognition [78], increases one’s persuasive power [74], and mobilizes followers [85]. The result is also in line with public voices that observe an increasing social acceptance of non-anonymous digital hate speeches [99].

This study also has practical implications. First, it can be expected that in the future, digital norm enforcement will intensify. The growing digital civil society adapts to the digital environment that transforms interactions. Social media offer great opportunities for individuals who have the intrinsic desire to enforce norms and contribute to the formation of latent interest groups. Second, the regularly demanded abolition of online anonymity and the introduction of real-name policies do not necessarily prevent online aggression in social media. Our view is in line with findings from a natural experiment in South Korea where the enacting of a Real Name Verification Law in 2007 only reduced aggressive comments for a particular user groups, but not for others [73]. There is, however, no doubt that the battle over online anonymity will intensify over time, particularly when aggressive norm enforcement by the civil society not only addresses low status, but increasingly high status, actors such as states or corporations.

This study has several limitations that should be kept in mind when interpreting the results. First, the findings are only generalizable to direct, explicitly abusive online aggression but not to indirectly formulated aggression such as cynicism. Also, while we qualitatively checked comments in our large dataset, it was not feasible to identify all comments. The amount of aggression in some comments may be therefore wrongly classified.

Second, in strict terms, the anonymity option of the petition design restricts the generalization of our findings to anonymity hidden from the internet community but not from the petition organizers. However, we consider the transferability to differing anonymity contexts as justified. This is because we do not refer to “true anonymity”, but to “relative anonymity”, i.e., exploring why spontaneous commenters decide between common options of (non-)anonymity offered for selection by most social media platforms. Achieving true anonymity, in contrast, is difficult anyway: although we recognize that there may be a minority of protesters providing pseudonyms and/or using Tor browsers to hide their identity from petition organizers, and their true anonymity, e.g. to national security agencies, may still not be granted. Consequently, we do not make any inferences on aggressive tendencies by “truly” anonymous commenters because we cannot trace true anonymity and we also expect that the greatest majority of commenters fall back on common (non-)anonymity options.

Third, the results may be not completely transferable to all other types of social media such as criticizing Amazon on Amazon’s Facebook fan page. Preexisting norms of cooperation within online petition platforms may lower the expected cost of sanctions. If an aggressive commenter is confronted with a diffuse mass of weak but supportive social ties, he more likely reveals his identity compared to a setting of oppositional ties that could rebuke him, or strong, influential ties that could control inappropriate language.

Fourth, the empirical design does not allow us to draw conclusions with respect to cause-and-effect interpretations. By alternative designs such as most suitably field experiments or intervention studies, it could be analyzed whether the decision to comment (non-)anonymously is indeed driven by social norm deliberations.

Fifth, more information about the protesters and norm violators would be desirable, such as information about their motivation or their socio-demographic characteristics. Exploring whether aggressive protesters differ from non-aggressive protesters on particular dimensions would be of interest here. In regard to aggressors’ motivations, another fundamental problematic remains: To what proportion does firestorm-like outrage reflect genuine public opinion? And to what extent does it represent auto-generated propaganda of political (ro-)bots or astroturfers, i.e., fake commenters paid by central coordination units such as political parties? Particularly if public actors increasingly give in to social pressures triggered by firestorms, distinguishing between democratic expression of a legitimate peer-group and a swarm of bots or astroturfers becomes increasingly difficult. Although we perceive the occurrence of bots within our petition data as low (because the lists of signatures finally given to the addressee of the petition had to include all names and home addresses of signers), this is a challenge that public actors and researchers are likewise confronted with.

While we introduced social norm theory to understand online aggression in social media, many open questions remain. A largely unexplored area is the effectiveness, or offline impact, of digital social norm enforcement. Are there digital accusations that are systematically often ill founded, or mostly justified? Also, beyond knowing that aggressive norm enforcers prefer non-anonymity, how often and under what circumstances do non-anonymous aggressive sanctions indeed help to mobilize other actors and to enforce social norms? Beyond this individual level of analysis, we also recommend focusing on the collective level. A first point is to study, in more detail, the role of selective incentives for (latent) group formation and aggressive acts in social media. Can alternative methods and applications confirm that latent groups aggress more often and mostly non-anonymously? Finally, we did not study the underlying dynamics of online firestorms. Under which circumstances, for example by enforcing which kind of norm and by which framing of sanctions, can online aggressors in social media mobilize other followers within hours?

To conclude, within the increasing penetration of digital media into public life, online aggression has become an effective tool for punishing norm violations and securing public goods. Academia and politics cannot ignore the social-political motivation of an aggressor when investigating online aggression in social media. Also, in the debate on how to legally handle online aggression, underlying social-political motivations must be taken into account in the tightrope walk between securing free expression of opinion and preventing hate speech. And finally, from an ethical perspective, altruistic punishments of norm violations to secure public goods are honorable. However, the question arises whether the aggressive means of punishments as obtained in firestorms are justified.

## Supporting Information

S1 Table Descriptive statistics and bivariate correlations. (DOCX)
