# Supplementary material for: Digital Social Norm Enforcement: Online Firestorms in Social Media
Source: PLoS One. 2016 Jun 17;11(6):e0155923. doi: 10.1371/journal.pone.0155923 (PMC4912099; doi:10.1371/journal.pone.0155923)
Supplement: S1 Table — (DOCX) [file pone.0155923.s001.docx]

**S1 Table. Descriptive statistics and bivariate correlations.**

| ID | Variable | Obs | Mean | Std.Dev. | Min | Max | 1 | 2 | 3 | 4 | 5 |
| --- | --- | --- | --- | --- | --- | --- | --- | --- | --- | --- | --- |
| 1 | Amount of online aggression (log) | 566052 | .19 | .40 | .00 | 2.77 |  |  |  |  |  |
| 2 | Anonymity | 566053 | .30 | .46 | .00 | 1.00 | -.01 |  |  |  |  |
| 3 | Intrinsic motivation (log) | 566053 | .28 | .51 | .00 | 3.09 | .03 | -.02 |  |  |  |
| 4 | Status of the accused (log) | 554782 | 2.06 | .32 | .69 | 2.40 | .04 | .06 | .02 |  |  |
| 5 | Controversy of accusation | 554782 | .58 | .22 | .00 | .75 | .04 | .05 | .05 | .13 |  |
| 6 | Accused is a natural person (vs. legal entity) | 554782 | .04 | .19 | .00 | 1.00 | .03 | -.01 | .02 | -.04 | .03 |
| 7 | Anonymity of social environment (log) | 542900 | 7.88 | 2.61 | .69 | 12.63 | -.03 | .02 | .01 | .00 | -.02 |
| 8 | Accusation is connected to a scandal | 554780 | .23 | .42 | .00 | 1.00 | .03 | .01 | .08 | .20 | .00 |
| 9 | Length of comment in words | 566053 | 20.10 | 12.98 | 1.00 | 57.00 | .11 | -.01 | .21 | -.05 | .01 |
| 10 | Number of protesters (log) | 554781 | 4.24 | 1.89 | .00 | 7.98 | .05 | .05 | .00 | .19 | .15 |
| 11 | Time of comment submission after petition opening | 566053 | 50343.15 | 61289.32 | .00 | 628470.00 | .05 | .07 | .07 | .27 | .21 |
| 12 | Scope of protest | 554782 | .70 | .28 | .00 | 1.00 | .09 | .05 | .02 | .19 | .18 |
| 13 | Success of the petition | 554782 | .16 | .36 | .00 | 1.00 | .05 | .05 | -.05 | .19 | .16 |
| 14 | Motives: Income/minimization of costs | 554782 | .39 | .49 | .00 | 1.00 | .01 | .06 | -.03 | .14 | .20 |
| 15 | Motive: Security/social order/traditional values | 554782 | .09 | .29 | .00 | 1.00 | .00 | .00 | -.05 | .00 | .01 |
| 16 | Motive: Independence/self-determination | 554782 | .26 | .44 | .00 | 1.00 | .03 | .05 | .04 | .26 | .22 |
| 17 | Motive: Increasing life quality and competence | 554782 | .40 | .49 | .00 | 1.00 | -.11 | -.05 | -.05 | -.19 | -.14 |
| 18 | Topic: Art/culture/education | 554782 | .23 | .42 | .00 | 1.00 | -.07 | -.02 | -.08 | -.16 | -.07 |
| 19 | Topic: Economics | 554782 | .12 | .32 | .00 | 1.00 | .01 | -.01 | -.08 | -.10 | .04 |
| 20 | Topic: Politics | 554782 | .08 | .27 | .00 | 1.00 | -.04 | .02 | .06 | .04 | .05 |
| 21 | Topic: Media | 554782 | .17 | .38 | .00 | 1.00 | .11 | .12 | -.04 | .36 | .21 |
| 22 | Topic: Environmental and animal welfare | 554782 | .08 | .27 | .00 | 1.00 | .06 | -.06 | .02 | -.10 | -.01 |

**S1 Table. Descriptive statistics and bivariate correlations (continuation).**

| ID | Variable | 6 | 7 | 8 | 9 | 10 | 11 | 12 | 13 | 14 | 15 | 16 | 17 | 18 | 19 | 20 | 21 |
| --- | --- | --- | --- | --- | --- | --- | --- | --- | --- | --- | --- | --- | --- | --- | --- | --- | --- |
| 7 | Anonymity of social environment (log) | -.02 |  |  |  |  |  |  |  |  |  |  |  |  |  |  |  |
| 8 | Accusation is connected to a scandal | .06 | .02 |  |  |  |  |  |  |  |  |  |  |  |  |  |  |
| 9 | Length of comment in words | .00 | .00 | -.03 |  |  |  |  |  |  |  |  |  |  |  |  |  |
| 10 | Number of protesters (log) | -.07 | -.02 | .20 | -.02 |  |  |  |  |  |  |  |  |  |  |  |  |
| 11 | Time of comment submission after petition opening | -.07 | .01 | .22 | -.03 | -.02 |  |  |  |  |  |  |  |  |  |  |  |
| 12 | Scope of protest | .01 | -.19 | .15 | -.04 | .21 | .18 |  |  |  |  |  |  |  |  |  |  |
| 13 | Success of the petition | -.08 | .00 | -.13 | -.04 | .24 | .15 | .22 |  |  |  |  |  |  |  |  |  |
| 14 | Motives: Income/minimization of costs | -.05 | .01 | -.07 | .03 | .29 | .20 | .04 | .20 |  |  |  |  |  |  |  |  |
| 15 | Motive: Security/social order/traditional values | .00 | -.02 | .05 | -.01 | -.11 | .02 | .02 | .05 | -.16 |  |  |  |  |  |  |  |
| 16 | Motive: Independence/self-determination | -.04 | .01 | .06 | -.04 | .19 | .21 | .19 | .36 | .01 | -.09 |  |  |  |  |  |  |
| 17 | Motive: Increasing life quality and competence | -.05 | .10 | -.05 | .03 | -.20 | -.20 | -.29 | -.14 | -.20 | -.07 | -.14 |  |  |  |  |  |
| 18 | Topic: Art/culture/education | -.08 | .08 | -.10 | .08 | .05 | -.21 | -.16 | -.17 | .18 | -.13 | -.22 | .29 |  |  |  |  |
| 19 | Topic: Economics | -.04 | -.10 | .00 | -.01 | -.12 | -.02 | .07 | .01 | -.15 | .22 | .03 | -.08 | -.19 |  |  |  |
| 20 | Topic: Politics | .09 | .00 | .17 | -.04 | .06 | -.01 | -.03 | -.07 | -.02 | -.05 | .11 | -.09 | -.16 | -.10 |  |  |
| 21 | Topic: Media | -.02 | .02 | .07 | -.06 | .25 | .42 | .31 | .46 | .38 | -.09 | .32 | -.27 | -.24 | -.16 | -.13 |  |
| 22 | Topic: Environmental and animal welfare | .03 | -.09 | -.12 | -.01 | -.15 | -.07 | .13 | -.05 | -.23 | -.07 | -.17 | -.14 | -.16 | -.11 | -.09 | -.13 |
